# Authentic Aroma and Compound-Specific Isotope Ratios (*δ*^13^C, *δ*^2^H) Profiles of Vanilla Pods (*V. planifolia* and *V. tahitensis*)

**DOI:** 10.3390/molecules30040825

**Published:** 2025-02-11

**Authors:** Long Chen, Purna Kumar Khatri, Mauro Paolini, Tiziana Nardin, Alberto Roncone, Roberto Larcher, Luca Ziller, Luana Bontempo

**Affiliations:** 1 Fondazione Edmund Mach (FEM), Via E. Mach 1, 38098 San Michele all’Adige, Italy; long.chen@fmach.it (L.C.); mauro.paolini@fmach.it (M.P.); tiziana.nardin@fmach.it (T.N.); alberto.roncone@fmach.it (A.R.); roberto.larcher@fmach.it (R.L.); luca.ziller@fmach.it (L.Z.); 2 Center Agriculture Food Environment, University of Trento, Via E. Mach 1, 38098 San Michele all’Adige, Italy; 3 Department of Agroecology, Aarhus University, Forsøgsvej 1, 4200 Slagelse, Denmark; purna.khatri@agro.au.dk

**Keywords:** vanillin, ethyl vanillin, volatile compounds, GC-MS/MS, GC-C/Py-IRMS

## Abstract

Stable isotope ratio analysis of carbon (*δ*^13^C) and hydrogen (*δ*^2^H) in vanillin has become a valuable tool for differentiating natural vanilla from synthetic or biosynthetic alternatives and for tracing its geographical origins. However, increasingly sophisticated fraud techniques necessitate ongoing refinement of analytical methods to ensure accurate detection. This study advanced the field by investigating minor volatile organic compounds as potential biomarkers for identifying botanical and geographical origins of vanilla products. Vanilla pods from the two main vanilla species, *V. planifolia* and *V. tahitensis*, were investigated using GC-MS/MS to analyze their aromatic profile and GC-C/Py-IRMS to determine compound-specific isotope ratios, providing, for the first time, detailed and authentic isotopic and aromatic profiles. Additionally, the potential natural presence of ethyl vanillin and its corresponding glucoside precursors—molecules commonly used as synthetic vanilla-scented fragrance agents in various foods and industrial products—was explored using UHPLC-HRMS. These findings contribute to robust methods for verifying vanilla authenticity, addressing flavor complexity and isotopic composition, and enhancing the detection of adulteration in vanilla-flavored products.

## 1. Introduction

Vanilla is among the most popular flavoring agents in various foods, beverages, pharmacies, and cosmetics [1,2]. Natural sources of the vanilla flavor are the pods of tropical orchid plants: *Vanilla planifolia* Andrews, *Vanilla tahitensis* J. W. Moore, and *Vanilla pompona* Schiede. *V. planifolia* is the most widely traded species, followed by *V. tahitensis* [3]. Although the characteristic vanilla aroma is mainly due to vanillin (4-hydroxy-3-methoxybenzaldehyde) [4,5], vanilla pods contain more than 60 aroma-active [6,7] and more than 200 volatile compounds [8]. These compounds contribute to vanilla extract’s complex and peculiar flavor, which synthetic products cannot exactly reproduce [4]. Among these compositions, 4-hydroxybenzaldehyde, 4-hydroxybenzoic acid, and vanillyl alcohol are some of those that are relatively more abundant [4,9]. Ultra-high Performance Liquid Chromatography-Electrospray/Quadrupole Time of Flight Mass Spectrometry (UHPLC-ESI/QTOF-MS) analysis also confirmed the presence of 260 phenolic compounds in vanilla extracts [8]. Factors such as geographical origin, harvest-year, and curing techniques may influence vanilla extract’s flavors and quality.

Vanilla extracts meet only a small fraction of global demand, with most of the market supplied by synthetic vanillin and the remainder by vanillin from natural precursors or microbial transformation [10]. Picking and curing processes are arduous and costly, and the harvest is less resistant to diseases and climate variations [2,11,12]. Consequently, vanilla extracts usually lead to premium prices [13], motivating fraudsters to substitute them with synthetic vanillin or lignin-derived vanillin but label them as vanilla extracts [14]. The synthetic vanillin may cost between 10–20 USD/kg, compared to natural vanilla that costs from a minimum of 1250 USD/kg to 4400 USD/kg. Because of the high price, the global market size was evaluated to be 627 million USD in 2022 [15].

Ethyl vanillin (3-ethoxy-4-hydroxybenzaldehyde) is less expensive than vanilla extracts but 3–4 times more intense and is also widely used as a flavoring agent to imitate or enhance the vanilla aroma [4,5,16,17,18]. Currently, all ethyl vanillin available in the global market is produced by chemical catalysis [19], despite reports indicating that it occurs naturally in trace amounts in Medallion^®^ “FL 16.30-128”, a new variety of strawberry [20]. Up to now, the presence of ethyl vanillin in vanilla extracts has not been reported, and thus the presence of this compound in commercial products is considered a fraudulent addition [16,21].

Quite a few analytical techniques can determine the authenticity of the vanillin or vanilla-flavored products, such as Ultraviolet-visible spectroscopy (UV-VIS) [22,23,24], spectrophotometric analysis with multivariate statistics and artificial neural networks [22,23,24], infrared and Raman spectroscopy [25]. Various analytical approaches based on chromatography were applied to differentiate natural products from synthetic ones [21,26,27,28]. Fraudsters are constantly seeking increasingly more sophisticated tricks to bypass technical checks, and new techniques are required to guarantee food authenticity.

Stable isotope ratio analysis has shown to be a promising tool to differentiate vanillin’s botanical sources and geographical origin and has been applied to quality control in food safety for decades [29,30]. The carbon isotope ratio (*δ*^13^C) is associated with photosynthesis and can identify the source of vanillin in question (C3, C4, CAM plants, and petroleum). Since vanilla is a CAM (Crassulacean acid metabolism) plant, the *δ*^13^C value of natural vanillin ranges from −15.5‰ to −22.0‰ [2,31,32]. In contrast, vanillin obtained from petroleum and lignin is more depleted in ^13^C (−36.2‰ to −24.9‰) and that from glucose (ex-glucose) is more enriched in ^13^C (around −12.5‰) [2,31,32,33]. More recently, new sorts of fraud arose to mimic the *δ*^13^C value of the natural vanillin by mixing synthetic/biosynthetic vanillin and ex-glucose vanillin [33]. In this case, *δ*^2^H of vanillin may provide additional information to help deal with the challenges of the overlapping *δ*^13^C values [34]. In addition, *δ*^2^H can also discriminate vanilla from different geographical origins because plants obtain hydrogen mainly from natural precipitation, which depends significantly on the local geographical parameters that influence the distribution of deuterium in the local water, such as altitude, latitude, distance from the sea, and level of rainfall [31,35]. Useful techniques currently being applied to verify the authenticity of vanilla extracts include site-specific natural isotope fractionation studied by nuclear magnetic resonance (SNIF-NMR) and quantitative ^13^C-NMR spectroscopy [36]. It is possible to measure the isotopic content even at individual atomic sites of the molecules by employing these techniques [30,35,37,38,39]. However, compared with isotope ratio mass spectrometry (IRMS), high quantity and high-purity samples are needed, which means laborious sample treatment and preparation before analysis [29].

According to the literature [39], vanillin is present in fresh vanilla pods as glucovanillin and is only released during curing. This finding led to the hypothesis that ethyl vanillin might similarly exist in fresh vanilla pods as glucoethyl vanillin, though it may remain unreleased after curing. The detection of vanilla flavor adulterated by the addition of ethyl vanillin has been achieved by quite a few techniques, including Fourier Transform Mid Infrared (MID-FTIR) spectroscopy and chemometrics [18], thin layer chromatography [5], headspace solid-phase microextraction coupled with Gas Chromatography-Mass Spectrometry (GC-MS) [28], and LC quadrupole linear ion trap mass spectrometry [40]. Ethyl vanillin can be synthesized following various processes, for example the safrole process and the lignin process. The lignin process is based on alkaline air oxidation of lignin containing sulphite liquor, which is a by-product of wood pulp processing in paper manufacture. To date, as far as we know, no studies have investigated the influence of added ethyl vanillin on the isotope analysis of vanillin.

Previous studies focused mainly on the principal components of vanilla flavor, such as vanillin [12,29,31,34,39,41,42,43]. However, minor substances that contribute to the particular vanilla flavor may also be useful biomarkers for verifying vanilla’s and related products’ botanical and/or geographical origin. It is, therefore, essential to identify other molecules that could meet these needs. Furthermore, extending the stable isotope ratio analysis to a larger number of volatile compounds may be more effective in checking for false declarations of vanilla extracts.

This study aimed to verify the presence of ethyl vanillin glucoside as the precursor of ethyl vanillin in vanilla pods, to identify additional molecular markers for determining the botanical and geographical origins of vanilla, and to assess whether the addition of ethyl vanillin influences the isotope ratios of vanillin. To achieve this, we developed and validated a cutting-edge analytical method, integrating GC-MS/MS and GC-combustion/pyrolysis-IRMS, to obtain a comprehensive chemical profile of volatile organic compounds (VOCs) and a more detailed compound-specific isotopic characterization of carbon and hydrogen in vanilla extracts (both *V. planifolia* and *V. tahitensis*).

Notably, this is the first study to investigate the presence of ethyl vanillin glucoside in vanilla pods, providing new insights into the natural pathways of biosynthesis in vanilla. For the first time, compound-specific isotope analysis has been applied to minor aromatic compounds in vanilla pods, opening new avenues for their use in authentication and botanical and geographical traceability of vanilla flavors. Furthermore, our study pioneers the application of isotope analysis to authentic vanilla extracts spiked with synthetic ethyl vanillin, enabling a more precise assessment of its impact on vanillin’s isotopic compositions. These groundbreaking advancements not only enhance the molecular understanding of vanilla but also provide innovative analytical tools for quality control, authentication, traceability, and fraud detection in the vanilla industry.

## 2. Results

### 2.1. GC-MS/MS Analysis

In the GC-MS/MS analysis, approximately 50 volatile organic compounds (VOCs) were detected and quantified (refer to Supplementary Table S1). Ethyl vanillin was absent in all analyzed vanilla extracts. Vanillin emerged as the predominant compound across all vanilla pod extracts, exhibiting relative abundances ranging from 35.1% to 81.9%, with an average concentration of 66.1%. Another major volatile compound, 4-hydroxybenzaldehyde, was detected at levels between 3.3% and 10.4% of the total VOCs identified and quantified. Table S1 summarizes the GC-MS/MS analysis for VOCs.

### 2.2. UHPLC-HRMS Analysis

**Figure 1:**
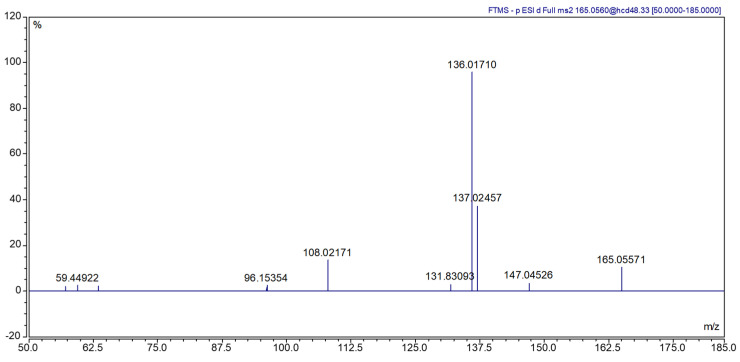
Fragmentation spectra of mass 165.0557.

Identification of ethyl vanillin glycosylated precursors was initially carried out using retention times (RT) and isotopic pattern of the extracting ion chromatogram (EIC) corresponding to the accurate mass (mass tolerance < 5 ppm) of the deprotonated molecules [M-H]^−^, *m*/*z* 327.1085, extracted from the full MS scan of the pure standard injection. Considering the low signal of the corresponding deprotonated ion, probably due to the sugar loss in HESI, the evaluation was performed by considering the ion corresponding to the aglyconic forms [M-H-C6H10O5]^−^, *m*/*z* 165.0557 (ethyl vanillin). Figure 1 shows the fragmentation spectra of *m*/*z* 165.0557.

The samples, however, showed numerous isomeric peaks with different fragmentations. To avoid false positives, a Parallel Reaction Monitoring (PRM) analysis was conducted. Finally, quantification was performed with ions at *m*/*z* 165.0557 while ions at *m*/*z* 136.0171 [M-H-C6H10O5-CH2-CH3]^−^ were used for identification. The ten in-house vanilla extract samples were analyzed with the PRM experiment and none showed the presence of the glycosylated precursor of ethyl vanillin.

### 2.3. Method Validation of GC-IRMS Analysis of Ethyl Vanillin

#### 2.3.1. Between-Run Precision

Between-run precision of the isotopic analysis was assessed for both *δ*^13^C and *δ*^2^H values. This assessment was conducted on commercial vanilla extract samples spiked with ethyl vanillin. The precision was expressed as the pooled relative standard deviation of the isotope ratios obtained from analysis of standard solutions on different days. Acceptable between-run precision was shown, as seen in Table 1, for carbon and hydrogen in either compound. Compared with carbon, hydrogen shows lower precision as observed in other studies [2,29,31,44].

#### 2.3.2. Within-Run Precision

The within-run precision was evaluated by performing 15 runs of standard solution in a single sequence on GC-IRMS then expressed as relative standard deviation of the 15 runs. The data in Table 2 show high within-run precision for *δ*^13^C and also for *δ*^2^H if we take into consideration the fact that the instrument has lower sensibility and stability in the analysis of hydrogen.

#### 2.3.3. Isotopic Fractionation During Solid Phase Extraction (SPE)

A series of analyses were conducted to evaluate possible isotopic fractionation caused by the SPE cartridge extraction. The differences in *δ*^2^H and *δ*^13^C values of the standard solution without SPE treatment and those of the standard solutions after SPE treatment were calculated. In both cases, GC-IRMS was used for isotopic analysis. The results revealed that the fractionation was smaller than or equal to the standard deviation and may be considered negligible (Table 3). The overall errors were calculated using the additive equation of error propagation, taking into consideration the isotopic fractionation during SPE and the within-run precision of the isotopic analyses.

#### 2.3.4. IRMS Analysis of Commercial Vanilla Extract Spiked with Ethyl Vanillin

**Figure 2:**
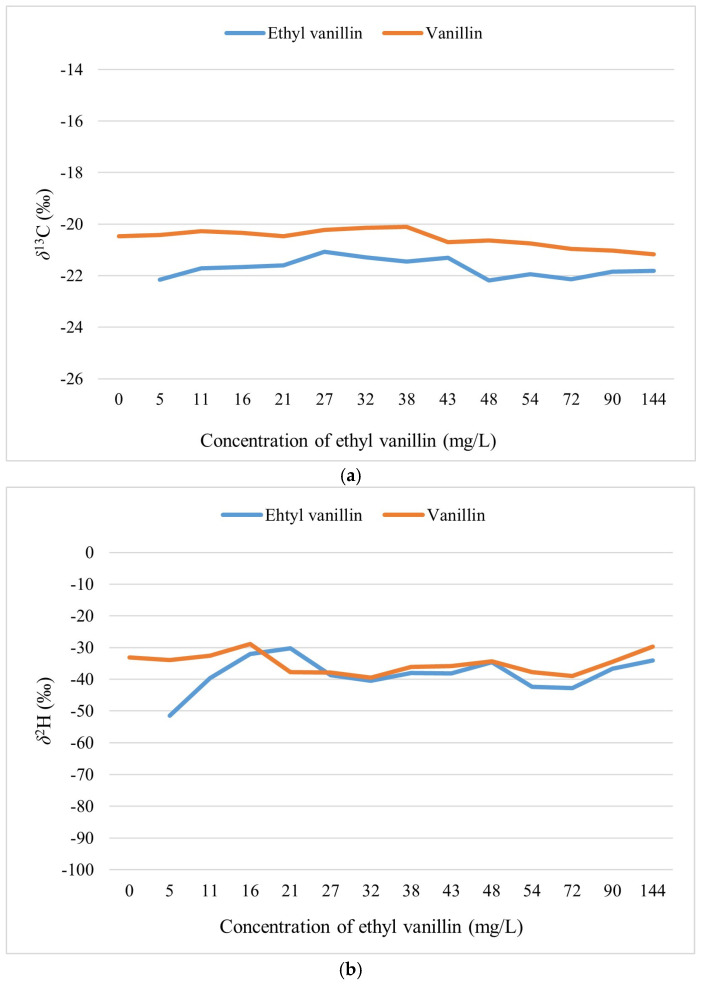
(**a**) *δ*^13^C values of vanillin and ethyl vanillin; (**b**) *δ*^2^H values of vanillin and ethyl vanillin.

The GC-IRMS analysis of the commercial vanilla extract containing added ethyl vanillin indicated a *δ*^13^C value of −20.5‰ for vanillin and −22.0‰ for ethyl vanillin, which aligns with typical values observed for C3 plants. These results may reflect the production process of the ethyl vanillin used in this study. As previously noted, ethyl vanillin can be synthesized through various pathways, with lignin as a key raw material. Additionally, the *δ*^2^H values of the ethyl vanillin, measured across multiple concentrations, ranged from −52‰ to −30‰. As seen in Figure 2, the concentrations of ethyl vanillin varied between 0 mg/L and 144 mg/L.

### 2.4. GC-IRMS Analysis of Vanilla Pods

As shown in Table 4, the *δ*^13^C values of vanillin of *V. planifolia* range from −20.5‰ to −19.1‰, while *V. tahitensis* exhibits a value of approximately −16.5‰, indicating that the latter is more enriched in the heavier carbon isotope. The *δ*^2^H values of vanillin range from −99‰ to −63‰ and vary across samples from different locations.

**Figure 3:**
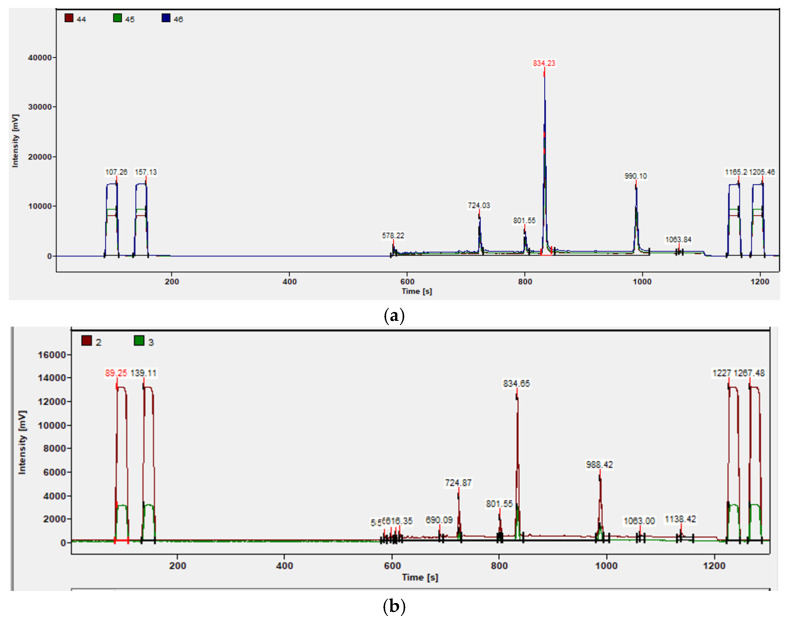
IRMS spectra of sample MAD1 where the peaks of the three selected minor compounds could be clearly identified: (**a**) *δ*^13^C and (**b**) *δ*^2^H.

In the in-house vanilla extracts, we identified three molecules as potential biomarkers for geographical and botanical discrimination of vanilla: homovanillic acid (RT = 724.03 s), 4-ethoxymethyl-phenol (RT = 801.55 s), and 4-hydroxy-3-methoxybenzyl alcohol (vanillyl alcohol, RT = 990.10 s). These compounds were separated from vanillin (RT = 834.23 s). These three molecules were selected due to their relatively high abundance, and they fall into a region without overlapping with other compounds. We attempted to evaluate the *δ*^13^C values of these minor components. Across various samples, the *δ*^13^C values ranged from −28.1‰ to −26.7‰ for homovanillic acid, −26.7‰ to −24.7‰ for 4-ethoxymethyl-phenol, and −31.7‰ to −28.9‰ for vanillyl alcohol; the *δ*^2^H values of varies between −109‰ and −68‰ for homovanillic acid, between −69‰ and −49‰ for 4-ethoxymethyl-phenol, and between −119‰ and −55‰ for vanillyl alcohol. Figure 3 shows the IRMS spectra of the sample MAD1 as an example.

## 3. Discussion

### 3.1. GC-MS/MS Analysis

**Figure 4:**
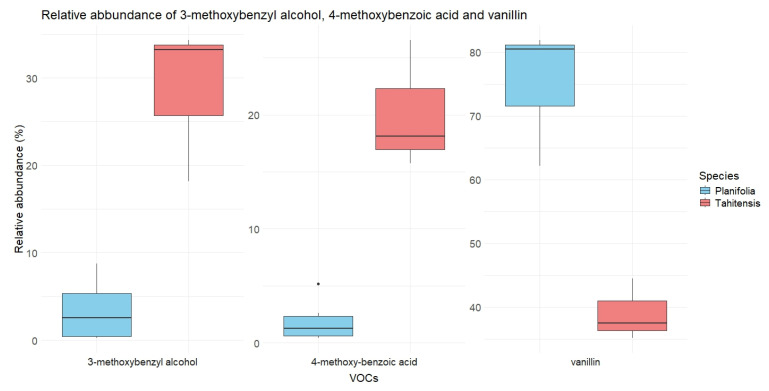
Boxplot of relative abundance (in %) of vanillin (*p* < 0.05), 3-methoxybenzyl alcohol (*p* < 0.05) and 4-methoxybenzoic acid (*p* < 0.05) in the two vanilla species.

The botanical differences between the two vanilla species can be seen in the boxplots (see Figure 4) of the relative abundance of vanillin, 3-methoxybenzyl alcohol, and 4-methoxybenzoic acid. Compared with *V. tahitensis*, *V. planifolia* contains more vanillin while the contents of the other two compounds are lower. Taking a closer look at the relative abundances of these compounds, it is not difficult to notice that vanillin takes only around 40% in *V. tahitensis*, while in *V. planifolia* it exhibits at least 60%. 3-methoxybenzyl alcohol, however, shows a much higher abundance in *V. tahitensis* (between 18% and 34%) than in *V. planifolia* (no more than 9%). 4-methoxybenzoic acid shows higher abundance in *V. tahitensis* (16–26%) than in *V. planifolia* (at most 5%). A student’s *t* test further confirmed the significant difference in the relative abundance of these compounds between the two species investigated.

Notably, certain compounds were detected in only a limited number of samples. For instance, anethole was identified in trace amounts exclusively in a vanilla pod from Mexico, while 2-phenylethyl acetate was present in only two of the Madagascar vanilla pods (MAD2 and MAD3). Furthermore, the relative abundance of some compounds displayed significant variability across samples. In sample MAD1, guaiacol, phenol, homovanillic acid, and 4-hydroxy-3-methoxybenzyl alcohol exhibited higher relative abundances compared to other samples. Variations were also observed within samples from the same geographic origin; for example, sample MAD1 had the lowest relative abundance of furfural but the highest levels of guaiacol and creosol. In contrast, sample MAD3 exhibited the highest abundance of 5-hydroxymethyl-2-furaldehyde. The observed differences in the relative abundance of VOCs, not only between different species (due to physiological factors) but also within the same species from the same country, suggest that additional factors—such as curing techniques, storage conditions, and crop-year—may have contributed to the variations identified in this study, as also noted by Belay et al. [4].

**Figure 5:**
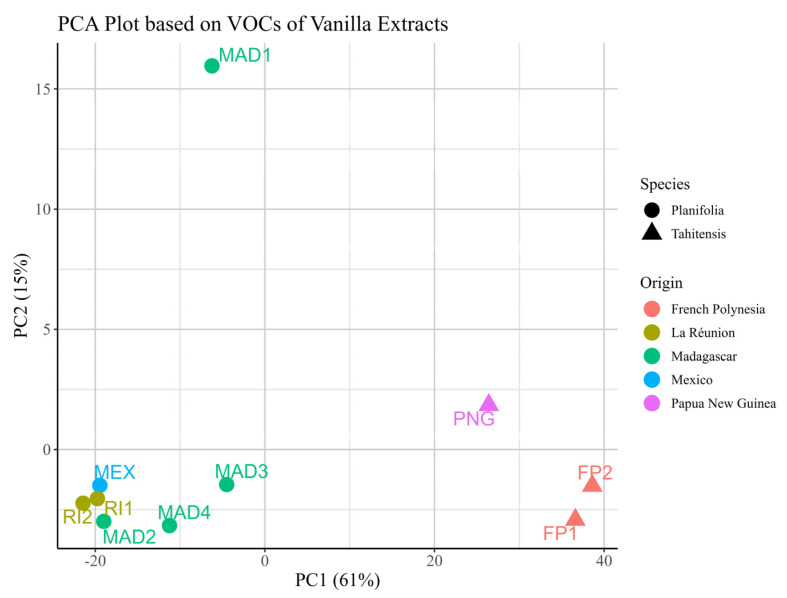
PCA plot of VOCs of different in-house vanilla extracts grouped by sample species and geographical origins.

Figure 5 shows the Principal Component Analysis (PCA) plots based on VOCs for grouping the samples by species and by geographical origins. As shown in Figure 5, the quantitative analysis of the VOCs in the vanilla extracts can distinguish different species of vanilla pods (*V. planifolia* and *V. tahitensis*). From the loading plot of the vanilla pods extracts (Supplementary Figure S1), it is not difficult to notice that vanillin, 3-methoxybenzyl alcohol, and 4-methoxybenzoic acid made the most contribution in PC1 while 4-hydroxy-3-methoxybenzyl alcohol, homovanillic acid, vanillin, and 3-methoxybenzyl alcohol contribute the most to PC2. Interestingly, vanillin and 3-methoxybenzyl alcohol made great contributions in both PCs. We can also notice that MAD1 stands out as an outlier among the samples from Madagascar. The loading plot (Supplementary Figure S2) specific to these samples highlights the main variables contributing to this distinction, with 4-hydroxy-3-methoxybenzyl alcohol, vanillin, and 3-methoxybenzyl alcohol identified as the top differentiating compounds. According to GC-MS/MS analysis, MAD1 has a significantly higher percentage of 4-hydroxy-3-methoxybenzyl alcohol and notably lower levels of vanillin and 3-methoxybenzyl alcohol. The high content of 4-hydroxy-3-methoxybenzyl alcohol may result from the reduction of vanillin, since the content of vanillin of MAD1 is relatively low. Possible reasons for this phenomenon might include variations in the curing procedures adopted by the producer. While the plot shows a clear separation of vanilla pods by species, differentiation based on geographic origin is less pronounced.

### 3.2. GC-IRMS Analysis of Vanillin and Ethyl Vanillin

As shown in Figure 2, the *δ*^13^C value of ethyl vanillin was stable within the uncertainty range of ±0.2‰ between 5 mg/L and 144 mg/L. Similarly, *δ*^2^H values of ethyl vanillin fall in the uncertainty range of ±3‰ at a concentration of at least of 21 mg/L. EA-IRMS and TC-IRMS were used to determine the reference values of *δ*^13^C and *δ*^2^H, respectively. The results obtained from GC-IRMS agreed with those obtained from EA-IRMS and TC-IRMS.

As already reported by Khatri et al. [44], below a certain concentration, the ionization potentials of the gas with different isotopic forms entering the ion source become unequal and could affect the isotopic value. Specifically, at concentrations below 21 mg/L, *δ*^2^H values of ethyl vanillin could not be accurately determined. On the other hand, a very high concentration may lead to the so-called “memory effect” of the instrument. This might be seen in the similar tendency of vanillin and ethyl vanillin at higher concentrations of ethyl vanillin in both *δ*^13^C and *δ*^2^H values. Samples at concentrations of 72 mg/L and above, for instance, show synchronized trends in *δ*^2^H values.

### 3.3. GC-IRMS Analysis of Vanilla Extracts

These two species of vanilla *V. planifolia* and *V. tahitensis* show statistically significant differences in *δ*^13^C value (α = 0.05, *p*-value < 0.05) of vanillin. These results are consistent with the findings of Hansen et al. and Greule et al. [2,29]. Research has shown that natural factors like light intensity, humidity, and the air’s CO_2_ content can influence how strongly a CAM plant relies on one photosynthetic pathway compared to the other one [45]. An early study found that CAM plants show different *δ*^13^C values depending on their growing conditions. Under high light intensity and high temperatures, their *δ*^13^C values resemble those of C4 plants, while under low light intensity and low temperatures, their *δ*^13^C values are closer to those of C3 plants [46]. Gassenmeier et al. [39] investigated the *δ*^13^C values of vanillin isolated from *V. planifolia* pods (Madagascar) prepared using different curing methods. Their study found a slight increase in *δ*^13^C values after the traditional curing, acid catalyzed hydrolysis of glucovanillin, and enzymatic release of vanillin. However, the reason for this increase remains unclear. All three processes produced final *δ*^13^C values ranging between −22.2‰ and −21.2‰, consistent with our results for *V. planifolia*. This suggests that the difference in *δ*^13^C values between the two species is likely due to physiological processes involved in carbon fixation and metabolism rather than post-harvest curing methods.

**Figure 6:**
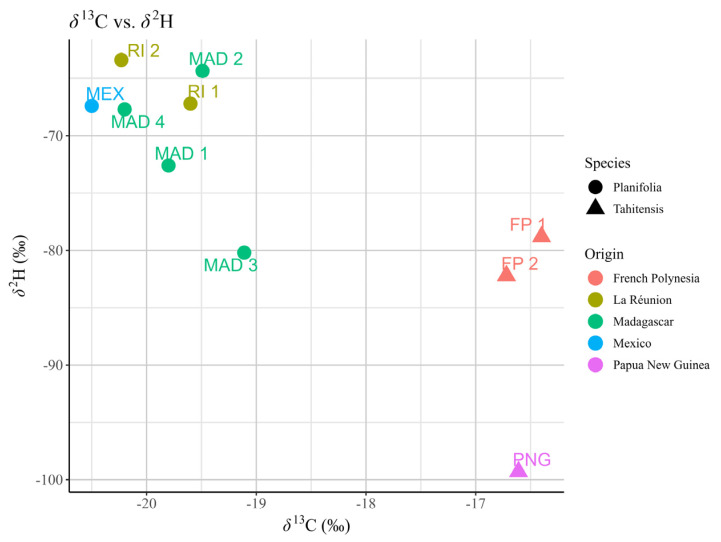
Scatter plot of *δ*^13^C and *δ*^2^H of vanilla extracts based on geographical origin and species.

Since precipitation is the primary source of hydrogen in plants, its *δ*^2^H values depend significantly on factors like longitude, altitude, and proximity to oceans or seas. Coastal and dry regions show more positive hydrogen delta values in plants due to isotopic differences in rainfall. Heavier isotopes precipitate near coasts, while lighter isotopes in inland precipitation reflect the movement of clouds [47,48]. Consequently, a plant’s *δ*^2^H value reflects its habitat. For example, *V. tahitensis* samples can be distinguished by their *δ*^2^H values: vanilla pods from French Polynesia are more enriched in deuterium than those from Papua New Guinea. Vanilla pods from Madagascar exhibit a wider range of *δ*^2^H values, with the sample labelled MAD3 being more depleted in deuterium than other Madagascan pods. The *δ*^2^H values measured in this study differ significantly from those reported by Hansen et al. [2]. These discrepancies may be caused by different harvest years, which led to different meteorological conditions. Another possible reason could be the different curing procedures and conservation conditions adopted by the producers, during which biochemical reactions took place leading to changes in isotopic compositions of deuterium. However, a more recent study by Perini et al. [31] found that the *δ*^2^H of vanilla pods might vary between −110‰ and −6‰, which aligns with our findings. Compared with *δ*^13^C, *δ*^2^H provides less insight into the vanilla species from which the vanillin was derived because of the difference observed [29]. The scatter plot (see Figure 6) of *δ*^13^C and *δ*^2^H values shows a clear separation of species.

**Figure 7:**
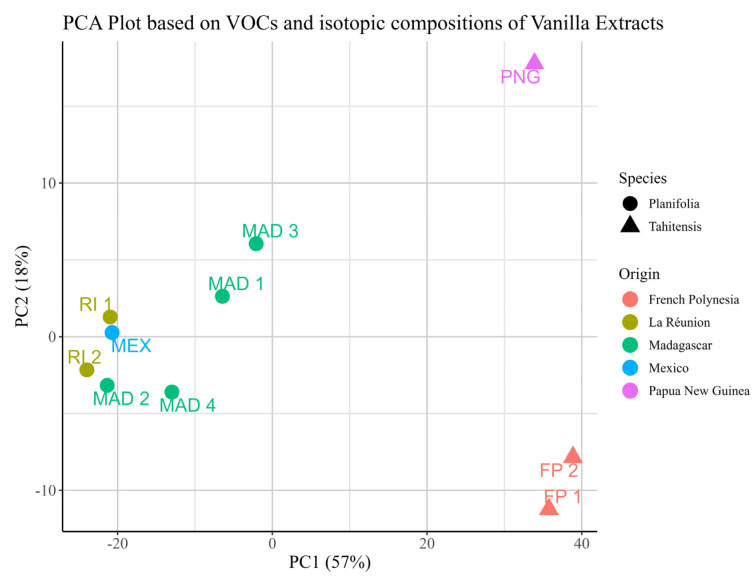
PCA plot based on VOCs, *δ*^13^C and *δ*^2^H values of different in-house vanilla extracts.

Since *δ*^2^H may suggest information about the geographical origins of the vanilla pods, an exploratory investigation by PCA (Figure 7) including these variables was performed. It is interesting to notice that the points representing the samples from Madagascar and those from La Réunion are classified in the same cluster in the PCA plot by regions; this could be explained by the fact that La Rénion and Madagascar are located in the same part of the world, and this might lead to the same climatic conditions in the two regions. In Figure 7, the sample from Mexico can be found in the same group as the ones from Madagascar. This may be caused by the fact that vanilla is mainly grown in the Totonacapan Puebla-Veracruz region in Mexico [49]. This region enjoys a tropical climate and is characterized by high temperatures, high humidity, and distinct wet and dry seasons, which is quite similar to the climatic conditions in Madagascar [41,50]. Considering this, it should not be surprising that the samples from these three countries and regions are grouped together.

## 4. Materials and Methods

### 4.1. Vanilla Samples

Ten vanilla samples native to Madagascar, Mexico, French Polynesia, Papua New Guinea, and La Réunion were collected. These samples are of two different species and were provided with quality remarks on the labels, as seen in Table 5.

### 4.2. Chemicals and Materials

The chemicals and materials used in this study are listed in Table 6.

Thirty-eight different compounds were used as reference standards (RS) for the identification of VOCs through GC-MS/MS analysis. These RS were purchased from various suppliers such as Sigma-Aldrich, Merck, Carlo Erba, and Fluka, with at least 95% purity (see Supplementary Table S2).

### 4.3. Commercial Vanilla Extract Preparation

To begin, 500 µL of commercial vanilla extract was diluted to 100 mL with Milli-Q water after the addition of 100 µL of internal standard (n-heptanol 500 mg/L) and increasing volumes of an ethyl vanillin solution (30 g/L) from 0 to 80% (*v*/*v*). Volatile compounds (VOCs) were extracted from the samples by adsorption on an SPE cartridge (Isolute ENV+, 1 g, 6 mL, Biotage, Sweden) previously conditioned with 15 mL of methanol and 20 mL of Milli-Q water. The sample was loaded onto the cartridge, which was washed with 20 mL of Milli-Q water, and the VOCs were then eluted with 30 mL of dichloromethane. The organic phase was dehydrated with anhydrous sodium sulphate and stored at −80 °C until analysis.

### 4.4. In-House Vanilla Extract Preparation

The extraction protocol was based on the ones described by other authors [2,11,32]. Briefly, the vanilla pods were cut longitudinally into two parts. Then, the seeds were removed carefully and gathered in a glass flask that had been previously weighed. Afterwards, the rest of the pods were cut into small pieces and collected in the same flask that was weighed again. The samples were then macerated with the solvent (Milli-Q water and ethanol 99.8%, 50:50 *v*/*v*). For every 15 g of vanilla sample, 100 mL of solvent was used. The extraction was performed at ambient temperature and lasted for 72 h with continuous stirring. When the extraction finished, the extracts were filtered through filter paper. The liquid phase was collected in another flask and stored at—20 °C for further treatments.

Subsequently, 500 µL of in-house vanilla extract was diluted to 50 mL with Milli-Q water after the addition of 100 µL of internal standard (mixture of 1-heptanol at 230 mg/L and ethyl-3-hydroxybutyrate at 1000 mg/L in ethanol/water 50:50). Volatile organic compounds were extracted as previously described in Section 4.3. To determine the isotopic compositions of minor compounds in the samples, the extracts were concentrated following the method of Paolini et al. [51], which was used for the analysis of VOCs in wine must, and it has been demonstrated that no isotopic fractionation had taken place during this procedure.

### 4.5. GC-MS/MS Analysis

VOCs analysis was performed using an Agilent Intuvo 9000 gas chromatograph coupled to a 7000C Triple Quadrupole mass spectrometer (Agilent Technologies, Santa Clara, CA, USA). The GC-MS/MS system was equipped with a PAL RSI 85 sampler (CTC Analytics AG, Zwingen, Switzerland) for the automated injection.

Separation was obtained by injecting 2 μL in split mode (1:5) into a 20 m DB-Wax Ultra Inert column (0.18 mm id × 0.18 μm film thickness; Agilent Technologies, Inc.) using He as carrier gas at a flow of 0.8 mL/min. The oven temperature was programmed starting at 40 °C for 2 min, raised to 55 °C by 10 °C/min, then raised to 165 °C by 20 °C/min, and finally raised to 240 °C by 40 °C/min and held at this temperature for 5 min. The MS transfer line temperature was set to 250 °C. Ionization was performed in electron impact (EI) mode at 70 eV; ion source and MS quadrupole temperatures were 230 °C and 150 °C, respectively. The mass spectra were acquired in full scan mode from 33 to 400 m/z.

Volatile compounds were identified by comparing their retention times and mass spectra with those of pure standards and with mass spectra from the NIST library (Version 2.4). Tentative identification of volatile compounds and/or confirmation of their identities was achieved by comparing the experimental linear retention index (LRI) with those reported in literature for columns of equal or equivalent polarity (see Supplementary Table S3) [52,53,54,55,56,57,58,59].

Agilent MassHunter WorkStation—Qualitative Analysis software (ver. B.08.00) was used for data analysis.

### 4.6. UHPLC-HRMS Analysis

To explore the presence of glucoethyl vanillin in vanilla extract, we performed an analysis using UHPLC-HRMS. Chromatographic separation was performed using an ultra-high performance liquid chromatograph (Thermo Ultimate R3000; Thermo Scientific, Sunnyvale, CA, USA), with an Acclaim^TM^ VANQUISH^TM^ Polar Advantage II column (2.1 mm × 150 mm, 2.2 μm particle size; Thermo Scientific, Sunnyvale, CA, USA) through a gradient of ACN in formic acid 100 mM with ammonium formate 20 mM, flow 0.4 mL/min, and running time 21 min. As regards high-resolution mass analysis, a tandem mass spectrometer (Q-Exactive^TM^; Thermo Scientific, Bremen, Germany) equipped with a heated electrospray source (HESI-II) was used in negative ion mode, acquiring spectra through full MS-data dependent MS/MS experiments (full MS–dd MS/MS), adapting the method proposed by Barnaba et al. [60] and in Parallel Reaction Monitoring (PRM) mode. Thermo Scientific^TM^ Dionex^TM^ Chromeleon^TM^ 7.3 Chromatography Data System (CDS) software was used for timing of the injection system chromatographic gradient, and for data processing and evaluation.

### 4.7. GC-IRMS Analysis

Stable isotope analysis was performed on Trace GC Ultra (Thermo Fisher Scientific, Waltham, MA, USA) coupled with an Isolink-IRMS (Delta V Isotope Ratio MS, Thermo Fisher Scientific) in parallel with a single quadrupole mass spectrometer (Thermo Fisher Scientific ISQ) for identifying the compounds in the samples. A mixture of synthetic vanillin and ethyl vanillin with known isotope ratios was used as standard at the beginning and end of the sequence. The MS transfer line was set at 250 °C and the ionization was performed in EI mode at 70V. The ion source temperature was 250 °C. The peak integration of vanillin and ethyl vanillin was processed in Isodat 3.0 (Thermo Fisher Scientific).

The separation for carbon isotope ratio analysis was obtained by injecting 2 µL of samples in splitless mode into a ZB-WAX capillary GC column (30 m × 0.32 mm × 0.50 µm, Phenomenex, Torrance, CA, USA) with He as the carrier gas (flow rate 2.0 mL/min). The initial temperature of the GC oven was set at 65 °C for 1 min then programmed to ramp by 20 °C/min to 250 °C, and it lasted for 12.70 min. The eluted samples were combusted at 1000 °C in a furnace reactor.

The separation for hydrogen isotope ratio analysis was obtained by injecting 2 µL of samples in splitless mode into a ZB-WAX capillary GC column (30 m × 0.32 mm × 0.25 µm, Agilent Technologies, Santa Clara, CA, USA) with He as the carrier gas (flow rate 2.0 mL/min). The initial temperature of the GC oven was set at 65 °C for 1 min, then programmed to ramp by 20 °C/min to 250 °C, lasting for 12.70 min. The compounds were pyrolyzed at 1420 °C in a high-temperature conversion (HTC) reactor.

The isotopic compositions were expressed as delta-notation (per mil) relative to the international standards VPDB (Vienna Pee Dee Belemnite) for *δ*^13^C and VSMOW (Vienna Standard Mean Ocean Water-Standard Light Antarctic Precipitation) for *δ*^2^H. The calculation was conducted according to the following equation [61]:

δrefEi/Ej,sample=REi/Ej,sampleREi/Ej,ref−1

where the *R(^i^E/^j^E, sample)* is the ratio of the number of atoms of the heavier isotope to the number of lighter isotopes in the sample and *R(^i^E/^j^E, ref)* is the ratio of the number of atoms of the heavier isotopes to the number of lighter isotopes in the reference material.

### 4.8. Statistical Analysis

In the present study, statistical analyses were conducted using R (version 4.4.0) to differentiate vanilla extracts from various regions and species.

## 5. Conclusions

This research study quantified more than 50 volatile compounds in different vanilla pods. As expected, vanillin was the predominant compound across all samples, with *V. planifolia* exhibiting higher vanillin concentrations, while *V. tahitensis* was enriched in 3-methoxybenzyl alcohol and 3-methoxy-benzaldehyde. The botanical classification of the two species could be observed on VOCs composition as well as based on the isotopic compositions of carbon and hydrogen. On the other hand, despite some differences, VOCs and isotope ratios of vanillin cannot completely discriminate the samples by their geographical origins.

A key breakthrough of this study was the confirmation—through UHPLC-HRMS analysis—that ethyl vanillin and its glucoside precursors were absent in genuine vanilla extracts, reinforcing that the detection of ethyl vanillin remains a reliable marker of fraud.

A significant methodological advancement was the development and validation of a GC-IRMS method for the compound-specific isotope analysis of authentic vanilla extract spiked with synthetic ethyl vanillin. This technique, applied for the first time to synthetic ethyl vanillin, provided precise and reliable isotope ratios of carbon and hydrogen, with validated accuracy and linearity. Concentrations of individual compounds (vanillin and ethyl vanillin) between 5 and 144 mg/L and between 21 and 72 mg/L, respectively, are required to produce reliable carbon and hydrogen isotope ratios. Importantly, GC-IRMS analysis confirmed that when properly separated, the presence of ethyl vanillin does not interfere with vanillin’s isotope ratios. Additionally, three newly identified potential molecular markers—homovanillic acid, 4-ethoxymethyl-phenol, and vanillyl alcohol—were characterized in both vanilla species. We then developed and validated a GC-IRMS method for determining the carbon and hydrogen stable isotope ratios of these compounds. The GC-IRMS method, applied for the first time to them, provided useful information about their stable isotope ratios, making them possibly capable of enhancing our ability to classify vanilla based on its botanical and geographical origins.

The first results of this preliminary investigation, combining both GC-MS/MS and GC-IRMS methods applied on ten extracted vanilla pods, resulted in interesting hints useful for differentiating vanilla extracts. In particular, different concentrations of some VOCs and the different isotopic compositions of individual VOCs demonstrated the capability to differentiate *V. planifolia* and *V. tahitensis*. The developed combined method could provide sufficient information to assess the botanical origin of vanilla extracts and to improve fraud detection in vanilla industry. Further studies based on analyzing more samples from different sources and origins are needed to confirm and refine these preliminary findings.

This work represents a significant step forward in the field of vanilla’s botanical authentication, not only advancing the molecular understanding of its chemical composition but also providing innovative analytical tools for quality control and regulatory enforcement.

## Figures and Tables

**Table 1:** Between-run precision evaluated using isotope ratios of vanillin ex lignin and ethyl vanillin.

	Ethyl Vanillin	Vanillin
*δ*^13^C	1.7%	0.7%
*δ*^2^H	12%	3%

**Table 2:** Within-run precision.

	*δ*^13^C (‰)		*δ*^2^H (‰)	
	Ethyl Vanillin	Vanillin	Ethyl Vanillin	Vanillin
Mean	−21.8	−28.5	−121	−174
SD	0.5	0.2	5	4
RSD	2.4%	0.6%	4%	3%

Notes: SD—standard deviation; RSD—relative standard deviation.

**Table 3:** Evaluation of isotopic fractionation induced by SPE cartridge.

Elements	Compound	Parameter	Standard	SPE	Fractionation	Overall Errors
*δ*^2^H	Ethyl vanillin	Mean	−192‰	−191‰	1‰	5‰
		SD	2‰	2‰		
		RSD	1%	1%		
	Vanillin	Mean	−228‰	−229‰	−1‰	4‰
		SD	2‰	1‰		
		RSD	1%	0.5%		
*δ*^13^C	Ethyl vanillin	Mean	−22.7‰	−22.7‰	0.0‰	0.5‰
		SD	0.5‰	0.1‰		
		RSD	1.7%	0.6%		
	Vanillin	Mean	−29.7‰	−29.9‰	−0.2‰	0.3‰
		SD	0.5‰	0.3‰		
		RSD	1.8%	1.2%		

Notes: Standard—standard solutions without SPE treatment; SPE—sample solutions after SPE treatment; Mean—average value of the standard solutions and sample solutions; SD—standard deviation; RSD—relative standard deviation.

**Table 4:** *δ*^13^C and *δ*^2^H values of vanillin in various vanilla extracts.

Sample Name	Geographical Origin	Species	*δ*^13^C (‰)	*δ*^2^H (‰)
FP1	French Polynesia	*Tahitensis*	−16.4	−79
FP2	French Polynesia	*Tahitensis*	−16.7	−82
MAD1	Madagascar	*Planifolia*	−19.8	−73
MAD2	Madagascar	*Planifolia*	−19.5	−64
MAD3	Madagascar	*Planifolia*	−19.1	−80
MAD4	Madagascar	*Planifolia*	−20.2	−68
MEX	Mexico	*Planifolia*	−20.5	−67
PNG	Papua New Guinea	*Tahitensis*	−16.6	−99
RI1	Réunion	*Planifolia*	−19.6	−67
RI2	Réunion	*Planifolia*	−20.2	−63

**Table 5:** Description of vanilla samples purchased.

Sample Number	Sample Name	Origin	Length (cm)	Species	Remarks
1	FP1	French Polynesia	18	*Tahitensis*	Gourmet quality
2	FP2	French Polynesia	18	*Tahitensis*	Gourmet quality
3	MAD1	Madagascar	16	*Planifolia*	High quality
4	MAD2	Madagascar	18	*Planifolia*	Grade A (gourmet quality)
5	MAD3	Madagascar	-	*Planifolia*	Glass tube (syrup sugar, pods 300 g/L, vanilla seeds)
6	MAD4	Madagascar	12	*Planifolia*	Grade B
7	MEX	Mexico	16	*Planifolia*	Gourmet quality
8	PNG	Papua New Guinea	16	*Tahitensis*	High quality
9	RI1	Réunion	13	*Planifolia*	Gourmet quality
10	RI2	Réunion	13	*Planifolia*	Gourmet quality

**Table 6:** Chemicals and materials used in this study.

Chemicals	Producer	Purity/Notes
1-heptanol	Merck, Darmstadt, Germany	≥99.5%
ethyl-3-hydroxybutyrate	Merck, Darmstadt, Germany	≥98.0%
Isolute ENV+ solid phase extraction (SPE) cartridges	Biotage, Uppsala, Sweden	1 g, 6 mL
Methanol	Merck, Darmstadt, Germany	≥99.9%
dichloromethane	Merck, Darmstadt, Germany	≥99.9%
anhydrous sodium sulphate	Merck, Darmstadt, Germany	≥99.0%
Hexane	Honeywell, Seelze, Germany	≥97.0%
Ethanol	Honeywell, Seelze, Germany	≥99.8%
ethyl hexanoate	Merck, Darmstadt, Germany	≥99.0%
synthetic ethyl vanillin	Sigma-Aldrich, Steinheim, Germany	≥98.0%
synthetic vanillin	in-house standard	with known delta values
vanillin ex lignin	in-house standard	with known delta values
commercial vanilla extract	Irca s.r.l, Gallarate, Italy	-
ethyl vanillin glucoside	Biosynth, Bratislava, Slovakia	-
Milli-Q water	in-house material	-

## Data Availability

Data will be made available on request.

## Supplementary Materials

The following supporting information can be downloaded at: https://www.mdpi.com/article/10.3390/molecules30040825/s1, Table S1: Volatile organic compounds (VOCs) quantified in various in-house vanilla extracts. Quantity expressed in percentages of total VOCs; Table S2: Standards used in the GC-MS/MS analysis for identifying VOCs present in in-house vanilla extracts; Table S3. Identification of volatile organic compounds by LRI (linear retention index), MS (mass spectra) and RS (reference standard). Calculated linear retention index (LRIa) on DB-Wax column relative to n-alkanes, LRI reported by NIST Mass Spectral Library (LRIb) and LRI reported in literature based on a DB-Wax columns or equivalent stationary phase (LRIc^)^ [52,53,54,55,56,57,58,59]; Figure S1 PCA loading plot of VOCs of different in-house vanilla extracts; Figure S2 PCA loading plot of VOCs of in-house vanilla extracts of vanilla originated from Madagascar.
