# Supplementary material for: Authentic Aroma and Compound-Specific Isotope Ratios (δ13C, δ2H) Profiles of Vanilla Pods (V. planifolia and V. tahitensis)
Source: Molecules. 2025 Feb 11;30(4):825. doi: 10.3390/molecules30040825 (PMC11858005; doi:10.3390/molecules30040825)
Supplement: Supplementary file 1 [file molecules-30-00825-s001.zip › molecules-3399965-supplementary.pdf]

Table S1: Volatile organic compounds (VOCs) quantified in various in-house vanilla extracts. Quantity expressed in percentages of total VOCs.

| Sample names | MAD1 | MAD2 | MAD3 | MAD4 | MEX | RI1 | RI2 | FP1 | FP2 | PNG |
|---|---|---|---|---|---|---|---|---|---|---|
| furfural | 0.005 | 0.162 | 0.242 | 0.096 | 0.100 | 0.049 | 0.058 | 0.012 | 0.012 | 0.166 |
| benzaldehyde | 0.007 | 0.005 | 0.012 | 0.006 | 0.006 | 0.008 | 0.010 | 0.004 | 0.003 | 0.006 |
| 5-methyl-furfural | 0.001 | 0.004 | 0.010 | 0.003 | 0.004 | 0.003 | 0.002 | 0.001 | 0.001 | 0.005 |
| furfuryl alcohol | 0.001 | 0.003 | 0.004 | 0.003 | 0.024 | 0.002 | 0.003 | 0.001 | 0.001 | 0.002 |
| anethol | 0.000 | 0.000 | 0.000 | 0.000 | 0.001 | 0.000 | 0.000 | 0.000 | 0.000 | 0.000 |
| guaiacol | 1.736 | 0.341 | 0.390 | 0.231 | 0.322 | 0.474 | 0.676 | 0.027 | 0.105 | 0.114 |
| cresol (4-methylguaiacolo) | 1.287 | 0.106 | 0.124 | 0.134 | 0.310 | 0.134 | 0.183 | 0.013 | 0.034 | 0.026 |
| maltol | 0.013 | 0.016 | 0.029 | 0.022 | 0.026 | 0.017 | 0.019 | 0.008 | 0.010 | 0.017 |
| phenol | 0.483 | 0.038 | 0.083 | 0.025 | 0.030 | 0.078 | 0.090 | 0.012 | 0.016 | 0.062 |
| p-cresol | 0.305 | 0.106 | 0.074 | 0.089 | 0.076 | 0.108 | 0.137 | 0.016 | 0.025 | 0.042 |
| 4-ethylguaiacol | 0.039 | 0.001 | 0.003 | 0.001 | 0.002 | 0.001 | 0.001 | 0.001 | 0.000 | 0.002 |
| 4-ethylphenol | 0.029 | 0.002 | 0.013 | 0.001 | 0.002 | 0.001 | 0.001 | 0.001 | 0.001 | 0.003 |
| syringol | 0.003 | 0.004 | 0.004 | 0.003 | 0.005 | 0.002 | 0.003 | 0.001 | 0.002 | 0.002 |
| 5-hydroxymethyl-2-furaldehyde | 0.091 | 1.103 | 5.462 | 0.588 | 1.421 | 0.642 | 0.647 | 0.028 | 0.083 | 2.071 |
| vanillin | 62.149 | 80.436 | 68.212 | 74.852 | 81.293 | 80.967 | 81.865 | 37.446 | 35.131 | 44.509 |
| acetovanillone | 0.079 | 0.141 | 0.082 | 0.106 | 0.133 | 0.103 | 0.124 | 0.019 | 0.020 | 0.039 |
| homovanillyl alcohol | 0.002 | 0.003 | 0.002 | 0.002 | 0.003 | 0.002 | 0.002 | 0.001 | 0.001 | 0.001 |
| syringaldehyde | 0.024 | 0.025 | 0.025 | 0.049 | 0.032 | 0.032 | 0.034 | 0.019 | 0.019 | 0.012 |
| eugenol | 0.005 | 0.002 | 0.005 | 0.002 | 0.004 | 0.001 | 0.001 | 0.001 | 0.001 | 0.034 |
| ethyl hexanoate | 0.001 | 0.000 | 0.005 | 0.001 | 0.001 | 0.001 | 0.001 | 0.000 | 0.000 | 0.000 |
| ethyl lactate | 0.089 | 0.015 | 0.075 | 0.048 | 0.279 | 0.006 | 0.004 | 0.003 | 0.002 | 0.017 |
| isovaleric acid | 0.037 | 0.040 | 0.021 | 0.054 | 0.046 | 0.038 | 0.039 | 0.007 | 0.007 | 0.021 |
| hexanoic acid | 0.015 | 0.034 | 0.208 | 0.033 | 0.019 | 0.037 | 0.043 | 0.008 | 0.006 | 0.017 |
| benzyl alcohol | 0.051 | 0.040 | 0.178 | 0.034 | 0.036 | 0.027 | 0.033 | 0.036 | 0.034 | 0.025 |
| 2-phenylethanol | 0.033 | 0.014 | 0.049 | 0.011 | 0.023 | 0.011 | 0.012 | 0.009 | 0.006 | 0.009 |

| | | | | | | | | | | |
|---|---|---|---|---|---|---|---|---|---|---|
| zingerone | 0.002 | 0.001 | 0.002 | 0.002 | 0.006 | 0.001 | 0.001 | 0.001 | 0.001 | 0.001 |
| 1-hexanol | 0.000 | 0.002 | 0.001 | 0.001 | 0.003 | 0.001 | 0.001 | 0.000 | 0.000 | 0.000 |
| ethyl dodecanoate | 0.000 | 0.001 | 0.003 | 0.001 | 0.001 | 0.000 | 0.001 | 0.000 | 0.000 | 0.001 |
| octanoic acid | 0.015 | 0.039 | 0.051 | 0.040 | 0.026 | 0.017 | 0.018 | 0.012 | 0.011 | 0.016 |
| nonanoic acid | 0.046 | 0.133 | 0.095 | 0.162 | 0.095 | 0.034 | 0.041 | 0.036 | 0.027 | 0.030 |
| decanoic acid | 0.008 | 0.008 | 0.029 | 0.011 | 0.011 | 0.014 | 0.010 | 0.006 | 0.008 | 0.013 |
| n-hexyl acetate | 0.000 | 0.002 | 0.001 | 0.001 | 0.003 | 0.001 | 0.001 | 0.000 | 0.000 | 0.000 |
| ethyl octanoate | 0.172 | 0.203 | 0.236 | 0.185 | 0.238 | 0.221 | 0.205 | 0.146 | 0.160 | 0.144 |
| ethyl decanoate | 0.003 | 0.010 | 0.006 | 0.010 | 0.008 | 0.007 | 0.003 | 0.004 | 0.003 | 0.003 |
| methyl salicylate | 0.003 | 0.004 | 0.001 | 0.003 | 0.001 | 0.003 | 0.003 | 0.000 | 0.001 | 0.000 |
| 2-phenylethyl acetate | 0.000 | 0.001 | 0.001 | 0.000 | 0.000 | 0.000 | 0.000 | 0.000 | 0.000 | 0.000 |
| butanoic acid | 0.002 | 0.004 | 0.008 | 0.002 | 0.003 | 0.003 | 0.004 | 0.002 | 0.001 | 0.002 |
| isobutyric acid | 0.004 | 0.004 | 0.004 | 0.003 | 0.006 | 0.004 | 0.003 | 0.003 | 0.003 | 0.002 |
| phenol, 2-methoxy- | 1.690 | 0.239 | 0.339 | 0.212 | 0.262 | 0.525 | 0.608 | 0.062 | 0.071 | 0.104 |
| 2-methoxy-5-methylphenol | 1.377 | 0.151 | 0.172 | 0.184 | 0.460 | 0.245 | 0.253 | 0.067 | 0.084 | 0.063 |
| benzaldehyde, 3-methoxy- | 0.190 | 0.168 | 0.616 | 0.393 | 0.252 | 0.164 | 0.115 | 3.480 | 3.223 | 1.691 |
| m-anisic hydrazide | 0.011 | 0.022 | 0.377 | 0.031 | 0.031 | 0.011 | 0.036 | 1.048 | 1.082 | 0.374 |
| ethanone, 1-(2-hydroxy-5-methylphenol)- | 0.171 | 0.358 | 0.367 | 0.391 | 0.324 | 0.242 | 0.189 | 0.565 | 0.775 | 0.481 |
| 3-methoxybenzyl alcohol | 0.360 | 2.700 | 8.724 | 8.012 | 2.533 | 0.535 | 0.242 | 34.306 | 33.238 | 18.184 |
| homovanillic acid | 4.500 | 0.484 | 1.025 | 1.458 | 1.636 | 1.270 | 0.714 | 0.082 | 0.103 | 0.119 |
| benzoic acid | 0.035 | 0.010 | 0.563 | 0.016 | 0.019 | 0.019 | 0.025 | 0.020 | 0.041 | 0.017 |
| phenol, 4-(ethoxymethyl)- | 2.959 | 1.491 | 1.481 | 1.975 | 0.952 | 4.144 | 4.402 | 0.157 | 0.181 | 0.389 |
| vanillin lactoside | 0.036 | 0.022 | 0.345 | 0.132 | 0.031 | 0.056 | 0.019 | 1.255 | 1.171 | 1.004 |
| 4-hydroxy-3-methoxybenzyl alcohol | 13.190 | 0.590 | 0.370 | 0.788 | 3.001 | 0.385 | 0.268 | 0.068 | 0.070 | 0.296 |
| benzoic acid, 4-methoxy- | 1.271 | 0.437 | 5.189 | 2.066 | 0.795 | 2.638 | 0.427 | 15.756 | 18.128 | 26.539 |
| benzaldehyde, 4-hydroxy- | 7.469 | 10.273 | 4.683 | 7.525 | 5.109 | 6.715 | 8.422 | 5.249 | 6.096 | 3.323 |

Table S2 Standards used in the GC-MS/MS analysis for identifying VOCs present in in-house vanilla extracts.

| **Compound** | **Supplier** | **Purity (%)** |
|---|---|---|
| furfural | Sigma Aldrich | ≥99 |
| benzaldehyde | Fluka | ≥99 |
| 5-methyl-furfural | Sigma Aldrich | ≥98 |
| furfuryl alcohol | Sigma Aldrich | ≥98 |
| anethol | Sigma Aldrich | ≥99 |
| guaiacol | Sigma Aldrich | ≥99 |
| creosol (4-methylguaiacol) | Sigma Aldrich | ≥98 |
| maltol | Sigma Aldrich | ≥99 |
| phenol | Sigma Aldrich | ≥97 |
| p-cresol | Sigma Aldrich | ≥99 |
| 4-ethylguaiacol | Sigma Aldrich | ≥98 |
| 4-ethylphenol | Sigma Aldrich | ≥99 |
| syringol | Sigma Aldrich | ≥95 |
| 5-hydroxymethyl-2-furaldehyde | Sigma Aldrich | ≥99 |
| vanillin | Sigma Aldrich | ≥99 |
| acetovanillone | Sigma Aldrich | ≥98 |
| homovanillyl alcohol | Sigma Aldrich | ≥99 |
| syringaldehyde | Sigma Aldrich | ≥98 |
| eugenol | Sigma Aldrich | ≥99 |
| ethyl hexanoate | Sigma Aldrich | ≥98 |
| ethyl lactate | Merck | ≥98 |
| isovaleric acid | Sigma Aldrich | ≥99 |
| hexanoic acid | Sigma Aldrich | ≥99 |
| benzyl alcohol | Carlo Erba | ≥99 |
| 2-phenylethanol | Sigma Aldrich | ≥99 |

| | | |
|---|---|---|
| zingerone | Sigma Aldrich | ≥98 |
| 1-hexanol | Sigma Aldrich | ≥98 |
| ethyl dodecanoate | Fluka | ≥98 |
| octanoic acid | Fluka | ≥99.5 |
| nonanoic acid | Sigma Aldrich | ≥97 |
| decanoic acid | Merck | ≥99 |
| n-hexyl acetate | Sigma Aldrich | ≥99 |
| ethyl octanoate | Sigma Aldrich | ≥99 |
| ethyl decanoate | Fluka | ≥99 |
| methyl salicylate | Sigma Aldrich | ≥99 |
| 2-phenylethyl acetate | Sigma Aldrich | ≥98 |
| butanoic acid | Carlo Erba | ≥99 |
| isobutyric acid | Fluka | ≥99.5 |

Table S3. Identification of volatile organic compounds by LRI (linear retention index), MS (mass spectra) and RS (reference standard). Calculated linear retention index (LRI[a]) on DB-Wax column relative to n-alkanes, LRI reported by NIST Mass Spectral Library (LRI[b]) and LRI reported in literature based on a DB-Wax columns or equivalent stationary phase (LRI[c]) [52-59].

| Compound | Identification methods | LRI[a] | LRI[b] | LRI[c] |
| --- | --- | --- | --- | --- |
| furfural | LRI, MS, RS | 1459 | 1461 | 1460 |
| benzaldehyde | LRI, MS, RS | 1524 | 1520 | 1520 |
| 5-methyl-furfural | LRI, MS, RS | 1568 | 1570 | 1573 |
| furfuryl alcohol | LRI, MS, RS | 1654 | 1661 | 1656 |
| anethol | LRI, MS, RS | 1815 | 1817 | 1826 |
| guaiacol | LRI, MS, RS | 1871 | 1860 | 1877 |
| creosol (4-methylguaiacol) | LRI, MS, RS | 1949 | 1956 | 1956 |
| maltol | LRI, MS, RS | 1968 | 1971 | 1966 |
| phenol | LRI, MS, RS | 2005 | 2000 | 1992 |
| p-cresol | LRI, MS, RS | 2071 | 2080 | 2073 |
| 4-ethylguaiacol | LRI, MS, RS | 2027 | 2032 | 2029 |
| 4-ethylphenol | LRI, MS, RS | 2182 | 2187 | 2185 |
| syringol | LRI, MS, RS | 2275 | 2273 | 2286 |
| 5-hydroxymethyl-2-furaldehyde | LRI, MS, RS | 2490 | 2496 | 2485 |
| vanillin | LRI, MS, RS | 2573 | 2568 | 2569 |
| acetovanillone | LRI, MS, RS | 2651 | 2640 | 2671 |
| homovanillyl alcohol | LRI, MS, RS | 2809 | 2806 | 2817 |
| syringaldehyde | LRI, MS, RS | 2912 | 2907 | 2906 |
| eugenol | LRI, MS, RS | 2159 | 2169 | 2162 |
| ethyl hexanoate | LRI, MS, RS | 1231 | 1233 | 1231 |
| ethyl lactate | LRI, MS, RS | 1342 | 1347 | 1339 |

| | | | | |
|---|---|---|---|---|
| isovaleric acid | LRI, MS, RS | 1658 | 1666 | 1667 |
| hexanoic acid | LRI, MS, RS | 1854 | 1858 | 1855 |
| benzyl alcohol | LRI, MS, RS | 1876 | 1870 | 1879 |
| 2-phenylethanol | LRI, MS, RS | 1907 | 1907 | 1903 |
| zingerone | LRI, MS, RS | 2785 | 2786 | 2786 |
| 1-hexanol | LRI, MS, RS | 1359 | 1355 | 1362 |
| ethyl dodecanoate | LRI, MS, RS | 1848 | 1843 | 1856 |
| octanoic acid | LRI, MS, RS | 2060 | 2060 | 2064 |
| nonanoic acid | LRI, MS, RS | 2168 | 2170 | 2165 |
| decanoic acid | LRI, MS, RS | 2271 | 2276 | 2269 |
| n-hexyl acetate | LRI, MS, RS | 1269 | 1273 | 1268 |
| ethyl octanoate | LRI, MS, RS | 1433 | 1435 | 1429 |
| ethyl decanoate | LRI, MS, RS | 1640 | 1639 | 1638 |
| methyl salicilate | LRI, MS, RS | 1776 | 1765 | 1782 |
| 2-phenylethyl acetate | LRI, MS, RS | 1820 | 1813 | 1821 |
| butanoic acid | LRI, MS, RS | 1621 | 1624 | 1630 |
| isobutyric acid | LRI, MS, RS | 1566 | 1570 | 1568 |
| 3-methoxyphenol | LRI, MS | 2094 | 2086 | - |
| 2-methoxy-5-methylphenol | LRI, MS | 1782 | 1789 | - |
| 3-methoxybenzaldehyde | LRI, MS | 1889 | - | 1895 |
| m-anisic hydrazide | LRI, MS | 1950 | - | 1948 |
| 1-(2-hydroxy-5-methylphenyl)ethanone | LRI, MS | 2190 | - | 2180 |
| 3-methoxybenzyl alcohol | LRI, MS | 2095 | - | 2100 |
| homovanillic acid | LRI, MS | 2998 | 3099 | 2992 |
| benzoic acid | LRI, MS | 2417 | 2414 | 2423 |
| 4-(ethoxymethyl)phenol | LRI, MS | 2539 | - | 2547 |
| vanillin lactoside | LRI, MS | 2667 | - | 2673 |
| 4-hydroxy-3-methoxybenzyl alcohol | LRI, MS | 2780 | - | 2787 |

| | | | | |
|---|---|---|---|---|
| 2-methoxybenzoic acid | LRI, MS | 2798 | - | 2792 |
| 4-hydroxybenzaldehyde | LRI, MS | 2962 | 2958 | 2960 |

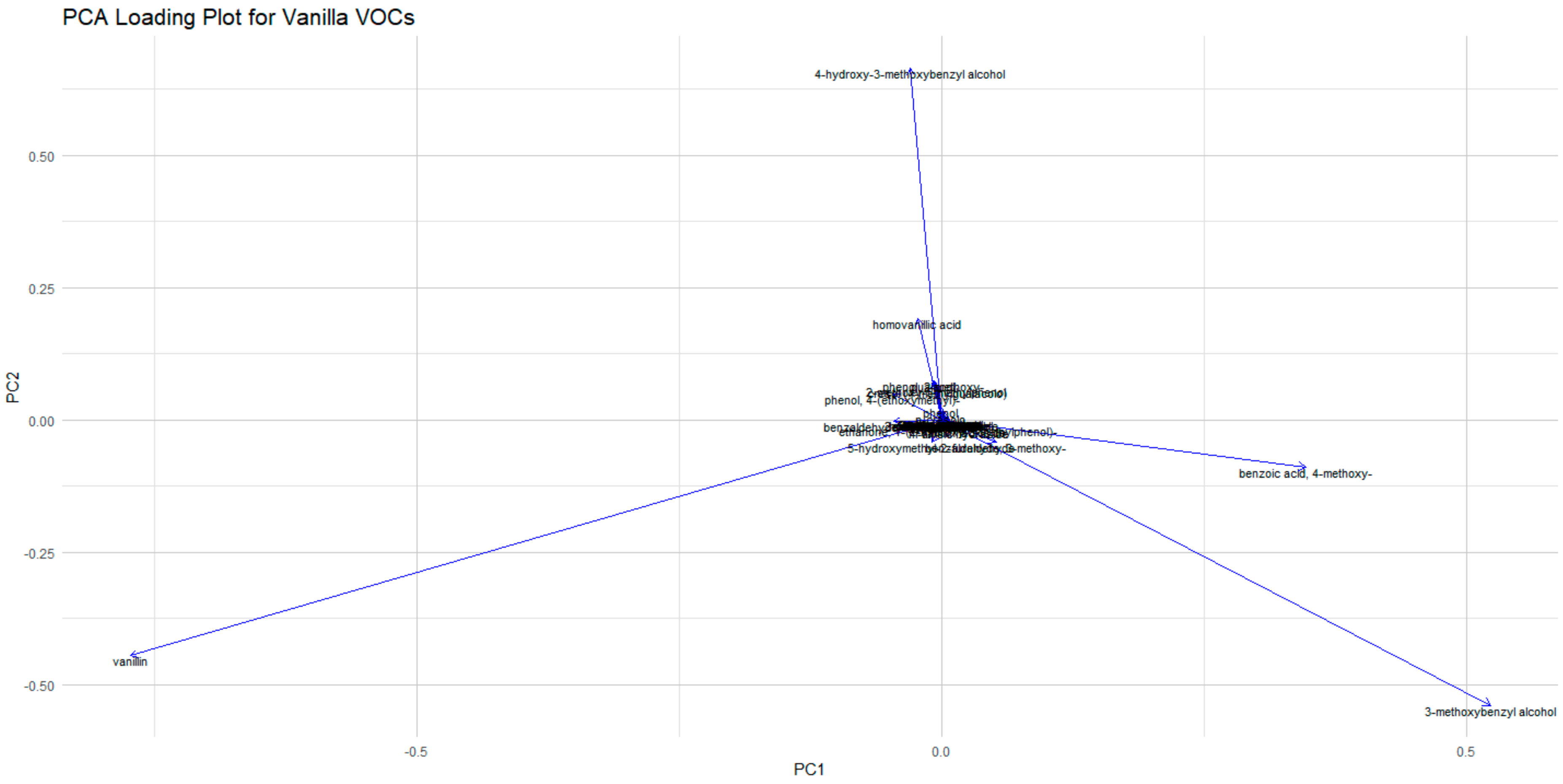


**Figure S1** PCA loading plot of VOCs of different in-house vanilla extracts.

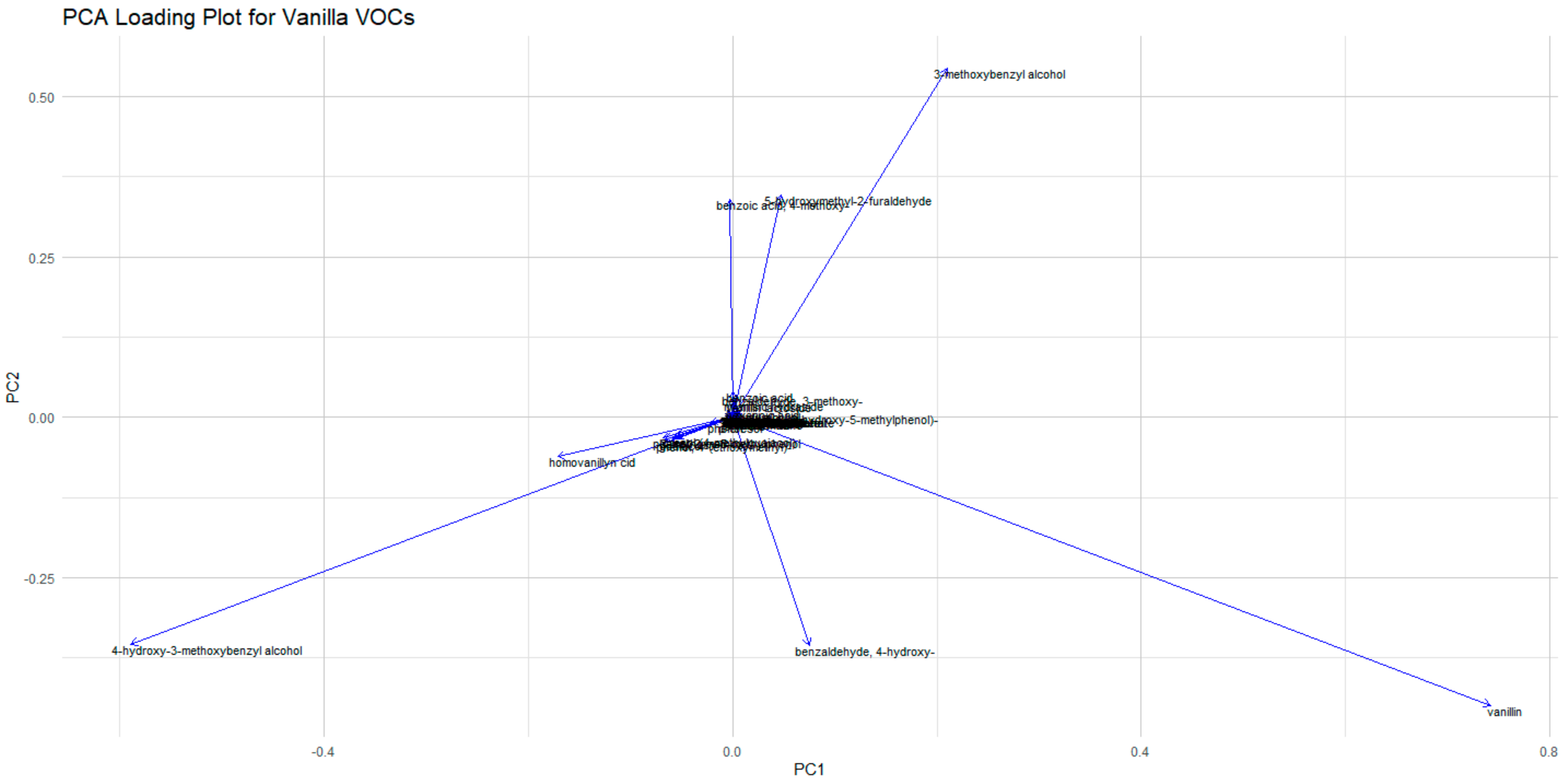


**Figure S2** PCA loading plot of VOCs of in-house vanilla extracts of vanilla originated from Madagascar.
